# Pathologically Entangled: Brain Trauma-Evoked ROS Imbalance Disrupts Kir Channel Function in Distant Peripheral Vessels

**DOI:** 10.1093/function/zqab021

**Published:** 2021-04-22

**Authors:** Nick Weir, Thomas A Longden

**Affiliations:** Department of Physiology, School of Medicine, University of Maryland, Baltimore, MD, USA

## A Perspective on “Traumatic Brain Injury Impairs Systemic Vascular Function Through Disruption of Inward-Rectifier Potassium Channels”

Traumatic brain injury (TBI) is a leading cause of morbidity, and systemic inflammation resulting from such an insult can contribute to a variety of further pathologies that negatively impact clinical outcomes.^1^ The circulatory system is a vast network that is tightly interwoven with the cells of every tissue and organ system, and thus can act as a vehicle conveying damage-causing agents from the primary site of injury in the brain to distant areas. Importantly, the vascular endothelium—which forms a continuous lining throughout the entire body that directly interfaces with the blood—is particularly vulnerable to damage resulting from circulating factors and cellular debris released in response to injury.

Kir2.1 is a member of the inward rectifier potassium (K^+^) channel family and is expressed by both capillary and arteriolar endothelial cells (ECs).^2^ This channel is activated by both extracellular K^+^ and membrane hyperpolarization^3^ and requires the membrane phospholipid, phosphatidylinositol-4,5-bisphosphate (PIP_2_) for its activity.^4^ As such, Kir2 channels play critical roles in mediating vasodilation to elevations of external K^+^, such as those that occur during brain activity or in working muscle, and in amplifying membrane hyperpolarization resulting from a broad range of other vasodilatory factors. The reliance of Kir2.1 on PIP_2_ for its activity opens the vasculature up to potential vulnerabilities post-trauma, as PIP_2_ degradation can occur in response to oxidative stress or systemic inflammation, potentially crippling vasoreactivity when it is needed most. Indeed, PIP_2_ degradation initiates a cascade of lipid-based metabolic events, and altered lipid metabolism is a poor prognosticator after trauma.

**Figure 1.**
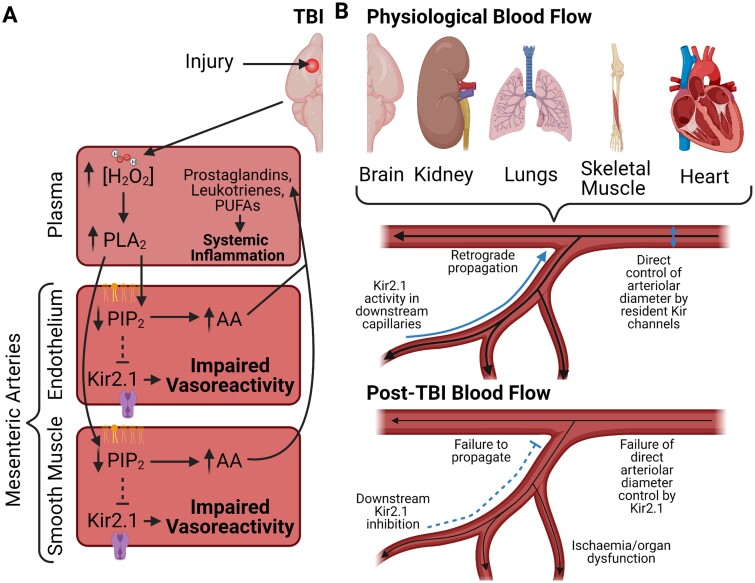
Schematic Overview of the Mechanism Defined by Sackheim et al. and the Implications for Blood Flow. (**A**) TBI elevates H_2_O_2_ which is then conveyed to the mesenteric arteries in the blood. H_2_O_2_ increases PLA_2_ activity and impairs Kir2.1 function, inhibiting vasoreactivity. The breakdown of PIP_2_ releases lipid byproducts from the vasculature into the plasma, resulting in disruption of the lipidome. (**B**) Kir2.1 plays a role in facilitating increased blood flow by directly controlling arteriolar diameter and propagating retrograde hyperpolarization from capillaries in a number of tissues. Loss of Kir2.1 functionality may lead to organ dysfunction in these tissues as a result of TBI.

In their most recent study, Sackheim et al. provide novel insight into the molecular basis underlying the pathological influence of TBI on the cells of the periphery (Figure 1). The authors present clear evidence that the function of mesenteric arteries is profoundly impaired by TBI and that the mediators of this dysfunction are blood-borne and indirectly impact Kir2.1 function. Further, they provide a convincing mechanistic link that connects brain trauma to functional impairment of these channels: damage to the central nervous system results in an increase in plasma hydrogen peroxide (H_2_O_2_), which activates phospholipase A_2_ (PLA_2_), dysregulating the lipidome. This in turn depletes PIP_2_ from ECs and smooth muscle cells (SMCs), which inhibits Kir2.1 and subsequently blunts vascular reactivity.

The group used a fluid percussion method to induce TBI prior to collecting ECs and SMCs of third- and fourth-order mesenteric arteries, in addition to plasma. Pressure myography was performed on mesenteric arteries and reduced constriction of these vessels to the Kir2.1 inhibitors Ba^2+^ and ML133 was observed, along with diminished responsiveness to 10 mM extracellular K^+^, while constriction to 60 mM K^+^ was preserved. These data confirm that TBI can have systemic effects on the vasculature and that Kir2.1 function is specifically impaired in this context. The conclusion that Kir2.1 was the ion channel responsible for loss of vasodilatory capacity within mesenteric arteries post-TBI was further reinforced using whole-cell patch-clamp electrophysiology to quantify Ba^2+^-sensitive currents in ECs and SMCs. As predicted, Kir2.1 currents were diminished in both of these cell types in vessels from animals subjected to TBI. As noted above, PIP_2_ is a known requirement for activity of the Kir2.1 channel. Remarkably, inclusion of a PIP_2_ analog in the intracellular solution in these experiments was capable of restoring the diminished Kir2.1 activity in ECs from TBI animals—an elegant and direct demonstration that the systemic vasculature was functionally impacted but not irreversibly damaged. Thus far, the group had established an effect distant from the site of injury and a mechanism for the inhibition of the channel, yet the mechanism of communication between the brain and periphery remained unresolved. Previously, Villalba et al.^5^ demonstrated that eNOS uncoupling occurs in the systemic vasculature after TBI, resulting in oxidative stress. Accordingly, they hypothesized that reactive oxygen species (ROS) imbalance could activate PLA_2_ and disrupt the lipidome, resulting in hydrolysis of vascular PIP_2_ and subsequent inhibition of Kir2.1. Further results confirmed a shift in the redox balance toward oxidation in the plasma and, using catalase to drive its decomposition, the authors confirmed that the primary elevated ROS was H_2_O_2_. Next, they hypothesized that PLA_2_ activity would be elevated as a result of this increase in oxidative stress, and measurements of enzymatic activity confirmed this. PLA_2_ lies upstream of a series of lipid metabolic reactions and thus, alterations to the activity of this keystone enzyme may disrupt the lipidome. In keeping with this notion, mass spectrometry revealed that the lipidome of mice afflicted with TBI is significantly altered, with several molecules downstream of PLA_2_ activity, such as prostaglandin A_2_, B_2_, and thromboxane A_2_, being upregulated. These data support the central role of PLA_2_ in the mechanism of Kir2.1 disruption while also raising further important questions regarding the knock-on effects resulting from the imbalances of these other signaling pathways on cell and tissue function.

The novelty and importance of this study lie in the unveiling of a detailed mechanism contributing to a highly prevalent pathology, which may in turn lead to the development of new therapeutic approaches to preserve or restore vascular function in TBI. Indeed, this research reveals a series of new possible points for therapeutic intervention: supplying antioxidants to reduce plasma H_2_O_2_, inhibition of PLA_2_, or the administration of PIP_2_ each might successfully restore Kir2.1 activity, and thus vasoreactivity. Furthermore, the mechanisms elucidated here may contribute to other conditions in which systemic inflammation or oxidative stress may occur.

The use of mesenteric arteries by the authors is a potent affirmation of the distal vascular effects of TBI, and other remote tissues may be of specific interest in light of this research. Indeed, Kir2.1 channels also play important roles in the juxtaglomerular cells of the kidney, in cardiomyocytes of the heart, in skeletal muscle, and within the placenta.^6^ Impairment of Kir2.1 may thus contribute to TBI-induced loss of function in these contexts also. Kir2.1 is also found in capillaries, where it is responsible for propagating a retrograde hyperpolarizing signal in the cerebral cortex during functional hyperemia to dilate upstream arterioles and increase blood flow.^7^ Similar roles for Kir2.1 have been observed in the heart^8^ and reported in skeletal muscle capillaries^9^—it is thus highly likely that capillary beds in other organs are also equipped to control upstream arterioles. In the brain, capillary-to-arteriole communication is also disrupted in TBI,^10^ but it remains to be seen whether TBI disrupts capillary Kir2.1 function in these other systems. Given that postinjury endothelial dysfunction is an independent predictor of multiorgan dysfunction,^1^ a quantitative analysis to determine if Kir2.1 function in these organs correlates with post-injury whole-organ dysfunction would be illuminating.

An additional question posed by this work is the broader scope of the intracellular damage induced by TBI systemically. While the authors focus specifically on Kir2.1-dependent vascular impairment, the capacity for widespread molecular dysfunction beyond that studied is tremendous. The key mediator of these changes is identified here as plasma H_2_O_2_, a nonpolar molecule that can cross the cell membrane and exert non-selective oxidative stress through systemic distribution through the vasculature. Within their mass spectrometry data, signaling molecules such as leukotrienes and prostaglandins are also dysregulated, suggesting cascades that could trigger immune responses or compromise vascular permeability. While Sackheim et al. uncover a specific mechanism that provides intriguing promise for novel therapeutic targets, there remains great potential for further clinically relevant discoveries as we further plumb the depths and breadth of TBI-induced systemic injury.

## Funding

Support for this work was provided by the NIH National Institute on Aging and National Institute of Neurological Disorders and Stroke (1R01AG066645-01 and 1DP2NS121347-01, to T.A.L), and the American Heart Association (19IPLOI34660108 to T.A.L).

## Conflicts of Interest Statement

None to declare.
